# Adequate vegetative cover decreases nitrous oxide emissions from cattle urine deposited in grazed pastures under rainy season conditions

**DOI:** 10.1038/s41598-018-37453-2

**Published:** 2019-01-29

**Authors:** Ngonidzashe Chirinda, Sandra Loaiza, Laura Arenas, Verónica Ruiz, Claudia Faverín, Carolina Alvarez, Jean Víctor Savian, Renaldo Belfon, Karen Zuniga, Luis Alberto Morales-Rincon, Catalina Trujillo, Miguel Arango, Idupulapati Rao, Jacobo Arango, Michael Peters, Rolando Barahona, Ciniro Costa, Todd S. Rosenstock, Meryl Richards, Deissy Martinez-Baron, Laura Cardenas

**Affiliations:** 1 International Center for Tropical Agriculture (CIAT), A.A, 6713 Cali, Colombia; 2 National Autonomous University of Nicaragua, Managua, Nicaragua; 3 National Institute of Agricultural Technology (INTA), Balcarce, Argentina; 4 National Institute of Agricultural Technology (INTA), Manfredi, Argentina; 5 Federal University of Rio Grande do Sul (UFRGS), Porto Alegre, Brazil; 6 The University of the West Indies, St. Augustine, Trinidad and Tobago; 7 Universidad Nacional de Colombia, sede Palmira, Colombia; 8 Universidad Nacional de Colombia, Bogotá, Colombia; 9 Universidad Nacional de Colombia, Medellin, Colombia; 10 The Colombian Corporation for Agricultural Research (AGROSAVIA), Villavicencio, Meta Colombia; 11 Present Address: Plant Polymer Research Unit, National Center for Agricultural Utilization Research, Agricultural Research Service, United States Department of Agriculture, 1815 North University Street, Peoria, IL 61604 USA; 12 Institute of Agriculture and Forestry Management and Certification (IMAFLORA), Estrada Chico Mendes, Piracicaba, Estrada Chico Mendes,185 13426-420 Brazil; 13 World Agroforestry Center (ICRAF), c/o INERA, Avenue des cliniques, Kinshasa, Democratic Republic of the Congo; 14 Rubenstein School of Environment and Natural Resources, University of Vermont, Burlington, VT 05405 USA; 15 Rothamsted Research, Sustainable Agriculture Sciences Department, North Wyke, Devon, EX20 2SB UK

## Abstract

A decline in pasture productivity is often associated with a reduction in vegetative cover. We hypothesize that nitrogen (N) in urine deposited by grazing cattle on degraded pastures, with low vegetative cover, is highly susceptible to losses. Here, we quantified the magnitude of urine-based nitrous oxide (N_2_O) lost from soil under paired degraded (low vegetative cover) and non-degraded (adequate vegetative cover) pastures across five countries of the Latin America and the Caribbean (LAC) region and estimated urine-N emission factors. Soil N_2_O emissions from simulated cattle urine patches were quantified with closed static chambers and gas chromatography. At the regional level, rainy season cumulative N_2_O emissions (3.31 *versus* 1.91 kg N_2_O-N ha^−1^) and emission factors (0.42 *versus* 0.18%) were higher for low vegetative cover compared to adequate vegetative cover pastures. Findings indicate that under rainy season conditions, adequate vegetative cover through proper pasture management could help reduce urine-induced N_2_O emissions from grazed pastures.

## Introduction

The livestock sector accounts for 46% of the agricultural gross domestic product of the Latin America and the Caribbean (LAC) region and grows at 3.7% annually^1^. Expanding livestock production is driven by a rapid increase in demand for cattle meat^2^. This increased demand for animal products together with the development of improved forage options to sustain higher levels of cattle productivity increases pressure on grasslands, the dominant cattle production systems of LAC, resulting in overgrazing and degradation of pastures^3^. According to Kwon^3^, an estimated 157 million ha (8% of total grazing area) of the grazing area in LAC is degraded. In Brazil half of the 80 million ha of introduced tropical pastures are estimated to be in some state of degradation as they have, among other symptoms, low soil cover^4^.

Cattle excreta deposited on grazed pastures is estimated to represent 16% of global anthropogenic nitrous oxide (N_2_O) emissions, a powerful greenhouse gas (GHG)^5^. About 75–95% of cattle ingested N is excreted in either urine or dung, which provides N-rich substrate for nitrification and denitrification^6,7^. Cattle urine patches can contain very high amounts of soluble N (equivalent to 500–1000 kg N ha^−1^), more than 2–3 times of the N uptake capacity of pastures^8^. Annually, about 1.5 Tg of total global anthropogenic N_2_O emissions (6.7 Tg N_2_O-N yr^−1^) are emitted from excreta produced by grazing cattle^9,10^ through both direct and indirect (from leached and volatilized excreta nitrogen) emissions. About 2% (0.7–6% uncertainty)^11^ of the nitrogen (N) in deposited urine is lost as N_2_O. Lower emission factors (EFs) (<0.7%), reported in other studies have been attributed to differences in climatic conditions, texture, soil moisture, and the N concentration in animal excreta^12^.

Pasture degradation may stimulate or constrain N losses. For example low vegetative cover, may reduce N sinks for deposited excreta and thus increase the vulnerability of N to loss through soil microbial processes and leaching. However, the low vegetative cover may also be associated with fewer plant root exudates and thus suppress microbial activity and N_2_O emissions^13^. On the other hand, overstocking and overgrazing without time for pasture recovery increases the risk of soil compaction - an indicator of pasture degradation. Soil compaction reduces soil porosity and pore continuity, decreases soil aeration, restricts plant growth and thus, consequently, increases soil N_2_O emissions from urine patches^14,15^. Soil acidification, which could also be an indicator of pasture degradation, has been shown to increase N_2_O emissions as acidic conditions generally reduce plant growth and inhibit N_2_O reductase enzyme activity which is responsible for transforming N_2_O to dinitrogen (N_2_)^16,17^.

Clearly, the effect of pasture degradation on N_2_O emissions from urine deposition can influence emission through multiple, often interacting, mechanisms and thus has produced contradictory results in the literature. Previous studies suggest that variations in soil N_2_O emissions from deposited urine patches in grazed pastures are driven by differences in several factors including ambient temperature^18^, urine volume and urine-N content^15,19^, soil drainage^20,21^, and soil moisture^22,23^. No previous studies have systematically explored the variation in urine-based soil N_2_O emissions associated with low vegetative cover in pastures.

Here we tested the hypothesis that N_2_O emissions from cattle urine deposited on grazed pastures with adequate vegetative cover are less intense than those from pastures with lower vegetative cover by measuring soil N_2_O fluxes from urine patches deposited on different pastures located at seven contrasting sites, spread across five countries in the LAC region during rainy season.

## Results

Soil texture at most of the study sites was similar in the low and adequate vegetation cover pastures with the exception of Balcarce (Argentina), Estelí (Nicaragua) and Taluma (Colombia) (Table 1). Soil pH values at the study sites ranged between 5.0 and 8.9, with acidic soils (pH < 6) at Taluma (Colombia), Rio Grande do Sul (Brazil), St. Augustine (Trinidad and Tobago) and neutral to basic soils at other sites. Soil bulk density at the study sites ranged between 0.6 and 1.6 g cm^−3^ and was generally similar between the low and adequate vegetative cover pastures at each study location. The largest differences in bulk density, soil organic carbon and soil organic nitrogen between low and adequate vegetative cover pastures at the Estelí location in Nicaragua (Table 1).

**Table 1:** Soil physical and chemical characteristics of the field sites of study areas.

Country	Location	Pasture Condition	Texture	pH	BD (g cm^−3^)	SOC (%)	SON(%)
Nicaragua	Estelí	AVC	Loam	6.4	1.1	3.0	0.2
		LVC	Clay	7.4	0.6	5.0	0.4
Colombia	Patía	AVC	Clay	6.4	1.5	2.2	0.2
		LVC	Clay	6.3	1.6	2.0	0.2
Colombia	Taluma	AVC	Clay loam	5.8	1.3	1.3	0.1
		LVC	Loam	5.2	1.5	1.3	0.1
Brazil	Rio Grande do Sul	AVC	Clay loam	5.0	1.6	1.4	0.1
		LVC	Clay loam	5.0	1.5	1.3	0.1
Argentina	Balcarce INTA	AVC	Sandy loam	7.5	1.0	3.5	0.3
		LVC	Sandy-clay-loam	8.9	1.1	3.3	0.3
Argentina	Manfredi INTA	AVC	Silt-loam	6.4	1.2	1.8	0.2
		LVC	Silt-loam	6.2	1.2	1.7	0.2
Trinidad and Tobago	St. Augustine	AVC	Sandy-Loam	5.0	*	*	*
		LVC	Sandy-Loam	5.1	*	*	*

AVC-Adequate vegetative cover; LVC- Low vegetative cover; BD: Bulk density, SOC: Soil organic carbon, SON: Soil organic nitrogen; *Missing data.

Air temperature and rainfall data for one week before and during the sampling dates are shown in Supplementary Fig. S1. The mean daily temperatures during this period ranged from 19 °C to 24 °C for Estelí (Nicaragua), 23 °C to 31 °C for Patía (Colombia), 27 °C to 29 °C for Taluma (Colombia), 17 °C to 25 °C for Rio Grande do Sul (Brazil), 15 °C to 26 °C for Balcarce (Argentina), 8 °C to 16 °C for Manfredi (Argentina) and 26 °C to 30 °C for St. Augustine (Trinidad and Tobago). Rainfall was recorded on less than 19 days during the N_2_O monitoring period, with the exception of the Trinidad and Tobago site which received 31 days of rainfall (Fig. S1).

**Figure 1:**
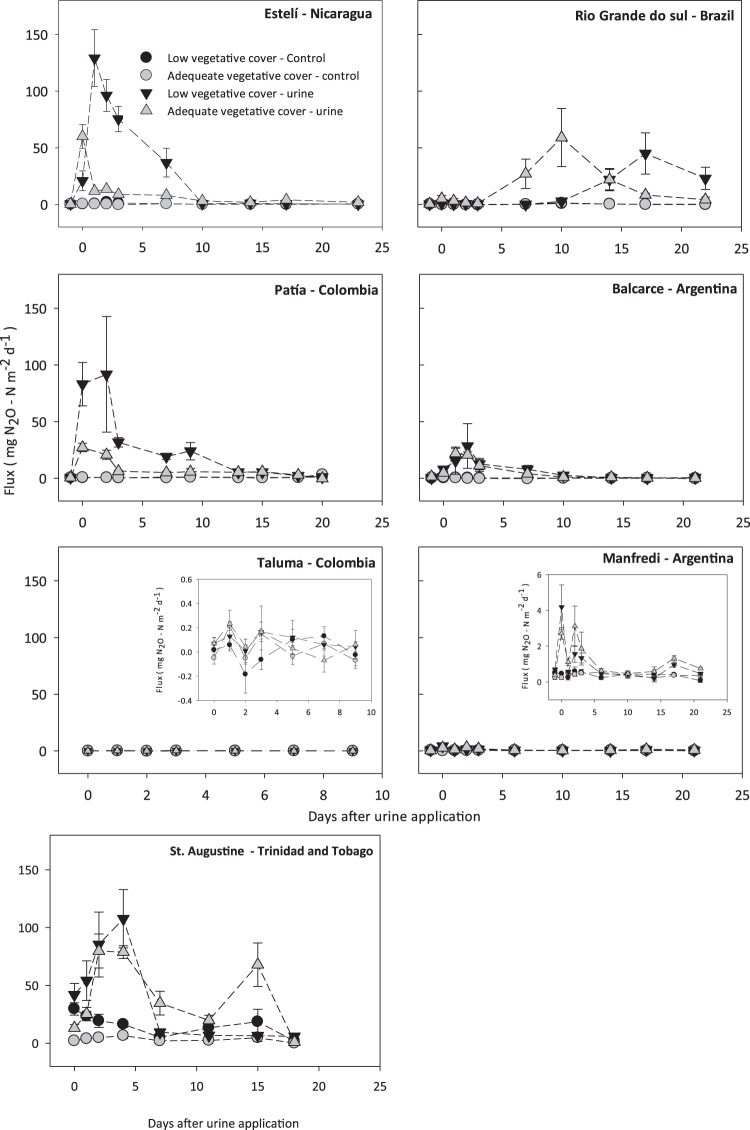
Soil N_2_O emission from two pasture conditions with cattle urine application at seven field sites in five Latin - America and the Caribbean countries. Error bars represent standard error of the mean. (AVC: Adequate vegetative cover, LVC: Low vegetative cover).

N_2_O emission peaks observed in LVC pastures tended to be higher than those in AVC pastures in 5 out of the 7 sites (Fig. 1; Table 2). However, the delayed N_2_O peaks observed at Rio Grande do Sul (Brazil) were higher in the AVC (59 ± 11 mg N_2_O-N m^−2^ day^−1^) compared to the LVC (45 ± 18 mg N_2_O-N m^−2^ day^−1^) pasture. The level of N_2_O emissions observed at Balcarce and Manfredi (Argentina) and Taluma (Colombia) sites were lower than 50 mg N_2_O–N m^−2^ day^−1^.

**Table 2:** Nitrogen inputs in applied urine, peak N_2_O emissions, cumulative N_2_O emissions.

Country	Location	Pasture Condition	Nitrogen in applied urine (kg N ha^−1^)	Peak N_2_O emissions (mg N_2_O-N m^−2^ d^−1^)	Cumulative N_2_O emissions (kg N_2_O-N ha^−1^)
Nicaragua	Estelí	LVC	464	129 (19)	5.82 (0.73)^a^
		AVC		60 (5)	1.85 (0.26)^b^
Colombia	Patía	LVC	789	92 (26)	3.85 (0.71)^a^
		AVC		27 (2)	1.41 (0.51)^b^
Colombia	Taluma	LVC	112	0.2 (0.2)	0.02 (0.01)^a^
		AVC		0.2 (0.1)	0.02 (0.005)^a^
Brazil	Rio Grande do Sul	LVC	619	45 (18)	4.59 (1.23)^a^
		AVC		59 (26)	3.01 (1.88)^a^
Argentina	Balcarce	LVC	1641	29 (10)	1.23 (0.57)^a^
		AVC		22 (2)	0.90 (0.14)^a^
Argentina	Manfredi	LVC	546	4.2 (0.5)	0.21 (0.01)^a^
		AVC		3 (0.5)	0.18 (0.02)^a^
Trinidad & Tobago	St. Augustine	LVC	*	107 (14)	7.49 (1.26)^a^
		AVC		80 (7)	6.00 (0.23) ^a^

AVC-Adequate vegetative cover; LVC- Low vegetative cover; number in parenthesis indicates standard error of mean (s.e.m). At each site values with the same letter for the cumulative N_2_O emission are not significantly different (P < 0.05).

**Figure 2:**
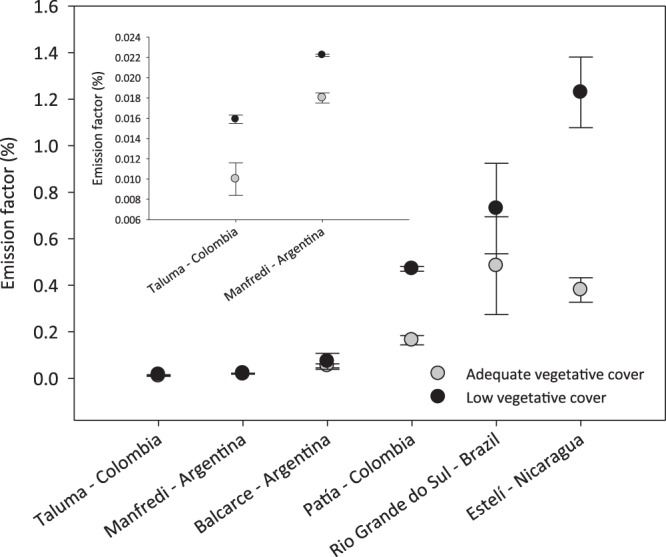
Emission factor (percent per applied nitrogen) from two pasture types (Adequate vegetative cover and Low vegetative cover) with the application of cattle urine. Error bars represent standard error of the mean.

The N content in applied cattle urine ranged from 112–1,641 kg N ha^−1^ (Table 2). Over a one-month period, the soil N_2_O emission factor of N in applied urine to soil ranged from 0.01 to 1.23%. The highest N_2_O emission factor values observed for the LVC pasture (1.23% of applied urine-N) and AVC (0.48% of applied urine-N) pasture were at Estelí (Nicaragua) and Rio Grande do Sul (Brazil), respectively. On the other hand, the lowest N_2_O emission factors for LVC (0.02% of applied urine-N) and AVC pasture (0.01% of applied urine-N) were both observed at the Taluma site in Colombia (Fig. 2). At the regional level, mean cumulative N_2_O emissions observed in the urine treatments ranged between 0.02 and 7.5 kg N_2_O–N ha^−1^. The highest cumulative N_2_O emissions for both treatments (LVC and AVC pasture) were observed at St. Augustine (Trinidad and Tobago) and Estelí (Nicaragua) and the lowest were observed at Taluma (Colombia) (Table 2).

At the regional level, mean N_2_O emission factors were significantly, at most 2.5 times, higher (P < 0.0002) in LVC (0.42 ± 0.19% SEM) than AVC (0.18 ± 0.08% SEM) pastures. Also at the regional level mean cumulative N_2_O emissions in the LVC (3.31 ± 1.09 kg N_2_O-N ha^−1^ SEM) were higher than those observed in the AVC (1.91 ± 0.78 kg N_2_O-N ha^−1^ SEM) pasture at the 10% level of significance (P = 0.08), based on results presented in Table 2. The N_2_O emissions at each individual site tended to be higher in LVC pastures than in the AVC pastures, with t-test detecting significant differences at the Nicaragua (Estelí) and Colombia (Patia) sites (Table 2). Rainfall for the measurement period (including data for the period of one week before commencing the monitoring campaigns) explained less of the variation in N_2_O emission factors in the LVC (66%) pasture compared to the AVC (88%) pasture (Supplementary Fig. S2). No clear effects of air temperature were observed on N_2_O emission factors.

## Discussion

The key finding of our study is that, at the regional level of LAC, N_2_O emission factors from cattle urine patches in grazed pastures are lower for AVC compared to LVC pastures during rainy season, suggesting the importance of adequate pasture improvement/management in mitigating soil N_2_O emissions. We did not have soil moisture data for all the sites to be able to compute the effects of water-filled pore space, which has been shown to be a major driver of N_2_O emissions^24,25^. The strong correlation observed between rainfall and the N_2_O emission factor suggests that soil moisture was possibly a key driver of N_2_O emissions in the current study. However, the fact that rainfall explained less of the variation in N_2_O emissions in the pasture with LVC compared to one with AVC may also imply that other factors, such as vegetative cover, are drivers of N_2_O emissions. Yet, interestingly, the steeper slope observed for the LVC (0.0086) compared to the AVC (0.0041) pastures suggests that urine deposited on LVC pastures is more vulnerable to high N_2_O losses when exposed to high rainfall. This may explain the significant differences between LVC and AVC pastures that were observed at Estelí (Nicaragua) and Patía (Colombia), where rainfall was high and the observed separation between the LVC and AVC pastures at the Rio Grande do Sul (Brazil) site where rainfall was also high.

High peaks of N_2_O emissions observed in LVC pastures compared to AVC pastures were likely due to lower plant N uptake from soil. Moreover, this may in part explain why AVC pastures generally resulted in lower net cumulative N_2_O emissions compared to degraded pastures. Despite the significant difference (P < 0.10) in net cumulative N_2_O emissions between LVC and AVC pastures, at a regional level, site-level comparisons showed that cumulative N_2_O emissions from LVC pastures were only significantly higher than those of AVC pastures in two sites, at Estelí (Nicaragua) and Patía (Colombia). The fact that soils within the LVC pastures at the Estelí site were more clayey than those under AVC pastures may have also contributed to the high net cumulative N_2_O emission in the former. Previous studies have reported higher N_2_O emissions from urine deposited on fine textured soils^26^. However, at Patía, where both the LVC and AVC pastures were on a clay soil observed differences suggest that despite the obvious influence of soil texture, pasture condition, based on vegetative cover is a driver of N_2_O emissions.

Low emissions of N_2_O observed at the two study sites in Argentina could have been due to the lower mean air temperatures. At the Balcarce location, mean air temperatures and the amount of N in applied urine were higher than at the Manfredi location. Low temperatures are known to reduce microbial activity and thus the rate of N transformation processes such as nitrification and denitrification in soil and, consequently, N_2_O production^27^. We assume that low temperatures may also be the cause of low net emissions and emission factors at the site in Brazil. It is important to note that the high soil pH (>7) at the Balcarce site, which would be expected to further increase with urine application may have also resulted in the inhibition of nitrification and high ammonia volatilization^28–30^. In addition, at the Balcarce site, the high urine-N levels may have resulted in microbial stresses with possible impacts on soil N transformation^31^ and thus contributed to the low N_2_O emissions and emission factors.

At the Taluma location in Colombia, the absence of a N_2_O emission peak following urine application may be because the frequency of measurements at this site was insufficient to capture the expected N_2_O emission spike. Several other studies using manual static chambers have reported having missed the N_2_O peak, due to the low temporal resolution^32–34^. This problem can be resolved by increasing the frequency of monitoring using manual static chambers or switching to automated chambers. Alternatively, the N content in applied urine was also the lowest at Taluma, which implies that N_2_O fluxes could have been limited by N substrate availability. In addition, the forage grass (*Brachiaria humidicola*) that was used at Taluma had high nitrification inhibition capacity^35–37^, which could have also contributed to the observed low N_2_O emissions.

Absence of significant differences, in N_2_O emissions, between LVC and AVC pastures at the St. Augustine (Trinidad and Tobago), Balcarce (Argentina), and Manfredi (Argentina) sites was possibly due to the fact that the spatial variation of vegetative cover of grass (soil cover) in the LVC (50–70%) pasture was high. As a result, local farmers based their classification on animal productivity differences which are influenced by both quantity (biomass) and quality (e.g. digestibility and crude protein content) of the forage on offer to animals. This further suggests that N_2_O emission differences between LVC pastures and AVC pastures are driven by differences in soil cover. With high plant density and greater plant vigour, we expect greater uptake of the urine-N by plants which could reduce the amount of N available for microbial transformations in soil such as nitrification and denitrification. It is therefore not surprising that when soil cover was high in the LVC pastures, there was no significant difference in N_2_O emissions with AVC pastures. This was however not the case for LVC pasture at the Patía (Colombia) site, which, though having similar soil cover (50–70%) showed significant differences between LVC and AVC pastures. This difference may be due to dissimilar vegetation types at the studied sites which would also affect N uptake and thus N availability for N_2_O emissions^38^.

The IPCC Tier 1 emission factor for urine deposited on grazed forages is 2% with an uncertainty range of 0.7–6%^11^. During this short-term study, several of the emission factors were below the uncertainty range of the IPCC Tier 1 emission factor. While this may be due to the short gas monitoring period (1 month), several other studies conducted under temperate conditions^39–42^, reported a similar range of emission factors (0.02–1.63%) as we observed under warm temperate or sub-tropical conditions in Argentina and Brazil (0.02–0.7%). Similarly, the range of emission factors reported from this study under the tropical conditions in Colombia, Nicaragua and Trinidad and Tobago (0.01–1.2%) are in agreement with the range of values that have been reported from studies conducted under tropical conditions^43,44^.

We conclude that in addition to the known effects of rainfall, temperature and the amount of urine-N, the pasture condition based on vegetative cover also influences N_2_O emissions from cattle urine patches. When pasture degradation is associated with a reduction in vegetative cover, N_2_O emissions are expected to increase. Therefore, better regional understanding of the state of pasture degradation is vital for a robust understanding of N_2_O emissions from cattle urine deposits. More importantly, these findings suggest that improving soil cover/pasture condition through adoption of appropriate grazing and nutrient management practices may contribute towards mitigating excreta-based soil N_2_O emissions from grazed pastures during the rainy season. We expect findings from this regional study to contribute towards reducing uncertainties in future assessments on the importance of improving grassland management to achieve global commitments, such as the Bonn Challenge, 20 × 20 initiative^45^ and the Paris Agreement^46^.

## Methods

The experimental plots were located at seven different sites in five countries in LAC spanning diverse climatic conditions and soil types (Supplementary Fig. S3 and Table S1). Rainfall is bimodal in Estelí (Nicaragua) with two seasons from May to June and August to November. In Patía (Colombia), the wet seasons occur during the period from March to May and September to November. Rainfall in St. Augustine (Trinidad and Tobago) is also bimodal with wet seasons occurring from June to August and October to November. At the rest of the study sites rainfall is unimodal with the main rains occurring during the period February to December and September to April at Taluma (Colombia) and the two Argentinean sites, respectively. During the monitoring period rainfall and air temperature data was collected at the nearest weather stations at each of the different study sites.

At each of the study sites, paired experimental plots were set-up on fields with grazed pastures that were classified as having either low vegetative cover (LVC) or adequate vegetative cover (AVC). Pairs of LVC and AVC pastures were not always available at the very same location, but were always less than 1 km apart. We used a qualitative approach including expert knowledge, farmer perceptions and an arbitrary ranking system based on soil cover to define pasture conditions based on vegetative cover using criteria that combined those used by Hollman^47^, Brown^48^ and McCormick and Lodge^49^. Specifically, through visual assessments by forage scientists^50^ we broadly described soil cover as follows: low vegetation cover (<70%) and adequate vegetation cover (>70%) pastures where soil cover is simply the proportion of soil covered by vertical projection of a plant canopy and vegetative biomass (Supplementary Table S2). Aboveground biomass data for the study sites were also obtained from historical records when available. In addition, we also used local farmer assessments to differentiate low and adequate vegetation cover pastures.

On each pasture (low vegetative cover or adequate vegetative cover), experimental plots were organized following a systematic experimental design^51,52^ with five replicates per treatment (Supplementary Fig. S4). The two treatments were urine application and a control-without urine application. The urine was applied to individual independent plots and so there are five replicates for each of the control *versus* urine treatment within each site^53^. Individual replicate plots (2 m × 2 m) were demarcated within each pasture condition for making measurements of soil properties and N_2_O emissions. To simulate grazing, grass in each plot was cut to approximately 5 cm sward height, seven days prior to the beginning of the gas and soil sampling.

Prior to starting the experiment, ten soil subsamples (0–10 cm) were separately collected from LVC and AVC pastures, using augers with 5 cm diameter, and combined to give one composite soil sample for each pasture condition. Collected soil was characterized for texture, pH, total carbon (C) and organic and inorganic N as described by Gee and Bauder^54^, McLean^55^, and Vogel^56^, respectively. A total of 20 cylindrical PVC static chamber bases (10 per treatment) were inserted at the center of each subplot to a depth of 5 cm, five days prior to the start of gas and soil sampling. For each treatment, chamber bases with an internal diameter of 25 cm and a height of 10 cm were distributed in five replicate plots. At each site, cattle urine samples (about 7 L) that were collected from at least 10 local dairy cows were pooled and, immediately, following setting aside a subsample for N analysis, 500 ml of the collected urine was applied to soils to simulate a urination event on soil within each static chamber base at a rate of 1.27 L urine/m^2^. Nitrogen concentration in urine was characterized in each of the study countries using the direct distillation method described by Hoogendoorn *et al*.^57^. Unfortunately, we were unable to quantify urine-N in Trinidad and Tobago as there are currently no laboratories that quantify N in animal urine samples.

Gas measurements were conducted from a total of 20 non – vented PVC static chambers (10 cm height and 25 cm diameter) fitted with two rubber septa (one for gas sampling and another for inserting a thermometer). On each sampling day, PVC chambers were fitted to the chamber bases and sealed with an air-tight rubber belt. Syringes (15 ml) fitted with hypodermic needles were used to collect four gas samples from each of static chambers following chamber closure and at 15, 30 and 45 minutes after chamber closure. Collected gas samples were transferred to pre–evacuated 10 ml headspace glass vials fitted with rubber butyl septa crimp caps. At each site, at least eight gas samples were collected between the months of November to December in 2015 for the localities of Patía (Colombia) and Rio Grande do Sul (Brazil), Estelí (Nicaragua) and St. Augustine (Trinidad and Tobago). For Taluma (Colombia) and Balcarce (Argentina) measurements were conducted between February to March (2016) and at Manfredi, Argentina in the month of May (2016). Sampling months were chosen to coincide with the wet seasons at each of the study sites. The use of non-vented chambers has been reported to cause bias in the flux estimates^58^. Yet, Davidson *et al*.^59^ reported the possibility of artefacts with both vented and non-vented chambers, making it difficult to know which chamber yielded the ‘true’ flux. Since similar non-vented chambers were used at all sites, the chambers did not affect observed differences between AVC and LVC pastures. However, the calculated N_2_O emissions factors may differ from those measured in other published studies that used vented chambers.

Gas sampling frequency was as follows: once before the application of urine, 1 hour after urine application, daily for the first three days following urine application, twice a week during the second and third week and once during last week of the experiment. Due to logistical challenges, less frequent measuring campaigns were done at the Taluma site in Colombia. Immediately after the gas sampling campaign, vials were sent to the Greenhouse Gas Laboratory at the International Center for Tropical Agriculture (CIAT) in Colombia, where N_2_O concentration was analyzed by gas chromatography (GC-2014 Shimadzu), within a month upon arrival. The daily N_2_O fluxes were calculated by regressing N_2_O emissions from each chamber on each sampling date against time in order to calculate the hourly flux which was then multiplied by 24 to determine the daily flux. Each calculated flux was corrected for temperature and barometric pressure according to Ideal Gas Law. Subsequently, cumulative fluxes were calculated from daily N_2_O fluxes by interpolation between measurement days^60^.

The N_2_O–N emission factor for urine patches was calculated according to Sordi *et al*.^61^:

$$EF( \% )=\frac{({N}_{2}O\mbox{--}N\,emitted)-({N}_{2}O\mbox{--}N\,control)}{N\,applied\,}\times 100$$

where EF is the emission factor, N_2_O–N_emitted_ and N_2_O–N_Control_ are the cumulative N_2_O emissions from urine or control patches over the 18 to 24 days monitoring period. N_applied_ represents the amount of N in the applied urine.

Statistical analyses were conducted using the PROC MIXED procedure of SAS^62^. Cumulative N_2_O fluxes and emission factors were, correspondingly, log and square-root transformed to achieve normality and obtain homogenous variances. To determine effects at the region level, cumulative N_2_O fluxes were analyzed using a split-plot ANOVA where the main plot was the pasture condition (LVC, AVC) and split-plot was the nitrogen levels (with and without urine application) and the blocking factor was the location and the main plot error term was pasture condition nested within location. In addition, at the regional level the emission factor variable was analyzed using a one-way ANOVA where the treatment effect was the pasture condition and the blocking factor was the location (site). At the individual sites we used the t-test analysis for testing differences in emission factors as influenced by pasture condition.

## Supplementary information

Supplementary information
