# Supplementary material for: Adequate vegetative cover decreases nitrous oxide emissions from cattle urine deposited in grazed pastures under rainy season conditions
Source: Sci Rep. 2019 Jan 29;9:908. doi: 10.1038/s41598-018-37453-2 (PMC6351538; doi:10.1038/s41598-018-37453-2)
Supplement: Supplementary file 1 — Supplementary information [file 41598_2018_37453_MOESM1_ESM.docx]

**Adequate vegetative cover decreases nitrous oxide emissions from cattle urine deposited in grazed pastures under rainy season conditions**

Ngonidzashe Chirinda^1*^, Sandra Loaiza^1^, Laura Arenas^1^, Verónica Ruiz^2^, Claudia Faverín^3^, Carolina Alvarez^4^, Jean Víctor Savian^5^, Renaldo Belfon^6^, Karen Zuniga^7a^, Luis Morales^7b^, Catalina Trujillo^1^, Miguel Arango^8^, Idupulapati Rao^1,9^, Jacobo Arango^1^, Michael Peters^1^, Rolando Barahona^7c^, Ciniro Costa Junior^10^, Todd S. Rosenstock^11^, Meryl Richards^12^, Deissy Martinez-Baron^1^, Laura Cardenas^13^

**Tables**

Supplementary Table S1. Soil and climatic conditions at seven field sites located in five Latin America and the Caribbean countries.

Supplementary Table S2. Description of low vegetation cover (LVC) and adequate vegetation cover (AVC) at seven field sites in five Latin America and the Caribbean countries. ** Forage mixtures and shrub; ***Trees; † Cruz^1^; †† da Trindade^2^

Supplementary Table S3. Soil mineral nitrogen dynamics observed at six field sites in six Latin America and the Caribbean countries

**Figure**

Supplementary Figure. S1. Mean, daily temperatures (°C) and rainfall (mm) during the course of the study.

Supplementary Figure. S2. Relationship between rainfall and emission factor in low and adequate vegetative cover plots.

Supplementary Figure. S3. Location of seven field sites across five countries in Latin America and the Caribbean ArcGIS 9.1.^3^

Supplementary Figure. S4. Schematic representation of the experimental design of nitrous oxide emissions and soil properties.

| **Country** | **Location** | **Mean temp.**  **(^0^C)** | **Geographical Coordinates** | **Soil order in the sites** | **Mean annual rainfall (mm)**  **2006–2015** | **Mean annual Temp. 2006 – 2015** | **IPCC Climate zone (2012)** |
| --- | --- | --- | --- | --- | --- | --- | --- |
| Nicaragua | Estelí | 23 | 13° 5’0”N  86° 21’0”W | Oxisol | 1302 | 23 | Equatorial, Monsoonal |
| Colombia | Patía | 23 | 2° 28’01.8”N 76°31’01”W | Mollisol | 3011 | 20 | Equatorial, fully humid |
| Colombia | Taluma | 20 | 2°13'34.14"N 4°21'54.72"W | Oxisol | 2581 | 26 | Equatorial, fully humid |
| Brazil | Rio Grande do Sul | 19 | 30^◦^05’S  51°  39´W | Typic Paleudult | 1595 | 20 | Warm temperate, fully humid, warm summer |
| Argentina | Balcarce | 14 | 37° 45’S 58°  18´W | Mollisol | 815 | 14 | Warm temperate, fully humid, cool summer |
| Argentina | Manfredi | 16 | 31° 50’S  63° 44’W | Mollisol | 801 | 17 | Warm temperate, winter dry, hot summer |
| Trinidad & Tobago | St. Augustine | 27 | 10°  63'71.24"N  61°  42'93.98"W | Inceptisol | 1792 | 26 | Equatorial, Monsoonal |

**Supplementary Table S1.**

| **Country** | **Location** | **Pasture Condition** | **Current vegetation type** | **Age of current vegetation type  (years)** | **Previous vegetation type** | **Soil cover**  **(%)** | **Biomass plots  (kg DM ha^-1^)** | |
| --- | --- | --- | --- | --- | --- | --- | --- | --- |
| Nicaragua | Estelí | AVC | *Andropogon gayanus, Guazuma ulmifolia***, Enterolobium cyclocarpo**** | 9 | Forest | 70-80 | 1040 |  |
|  |  | LVC | *Paspalum notatum* | 25 | Pasture | <30 | 520 |  |
| Colombia | Patía | AVC | *Brachiaria hybrid Mulato II, Brachiaria brizantha and Megathyrsus maximus* | 3 | Cotton | > 80 | 2500 |  |
|  |  | LVC | *Canavalia brasiliensis and Dichanthium aristatum* | 3 | Cotton | 50-70 | 1500 |  |
| Colombia | Taluma | AVC | *Brachiaria humidicola* | 5 | Maize | 70-80 | 1000 |  |
|  |  | LVC | *Brachiaria humidicola* | > 20 | Pasture | 30-50 | 650 |  |
| Brazil | Rio Grande do Sul | AVC† | Grasses: *Axonopus affinis, Paspalum notatum, Andropogon lateralis G, Piptochaetium montevidense, Sporobolus indicus.* Non-grasses: *Eryngium, Baccharis* and *Vernonia*** | Unknown | Unknown | 70-80 | 1864†† |  |
|  |  | LVC † | Grasses: *Paspalum notatum, Andropogon lateralis G, Piptochaetium montevidense, Sporobolus indicus.* Non-grasses: *Eryngium, Baccharis* and *Vernonia*** | Unknown | Unknown | 50-70 | 685†† |  |
| Argentina | Balcarce | AVC | *Thinophyrum ponticum + Festuca arundinacea Schreb cv. Taita* | 5 |  | 70-80 | 2500 |  |
|  |  | LVC | *Thinophyrum ponticum + Distichlis sp +* Cynodon dactylon | 15 |  | 50-70 | 750 |  |
| Argentina | Manfredi | AVC | Alfalfa *(Medicago sativa)* | 2 | Soybean | 70-80 | 2950 |  |
|  |  | LVC | Alfalfa *(Medicago sativa)* | 2 | Soybean | 50-70 | 1050 |  |
| Trinidad & Tobago | St. Augustine | AVC | *Brachiaria mutica* | > 20 | Forest | >80 | 2500 |  |
|  |  | LVC | *Mixed shrub* | > 20 | Forest | 50-70 | 850 |  |

**Supplementary Table S2.**

| **Country** | **Location** | **Treatment** | **Days after urine application** | **NH_4_^+^** | | **NO_3_^-^** |
| --- | --- | --- | --- | --- | --- | --- |
|  |  |  |  | *_________mg. kg soil^-1^__________* | | |
| Nicaragua | Estelí | LVC | 1 | 43.07 | 27.20 | |
| Nicaragua | Estelí | LVC | 3 | 44.90 | 37.81 | |
| Nicaragua | Estelí | LVC | 8 | 54.43 | 16.89 | |
| Nicaragua | Estelí | LVC | 15 | 61.51 | 12.36 | |
| Nicaragua | Estelí | LVC | 24 | 24.12 | 36.11 | |
| Nicaragua | Estelí | AVC | 1 | 36.27 | 39.38 | |
| Nicaragua | Estelí | AVC | 3 | 38.84 | 49.61 | |
| Nicaragua | Estelí | AVC | 8 | 38.02 | 41.28 | |
| Nicaragua | Estelí | AVC | 15 | 22.07 | 44.93 | |
| Nicaragua | Estelí | AVC | 24 | 44.05 | 21.67 | |
| Colombia | Taluma | LVC | -1 | 97.07 | 0.00 | |
| Colombia | Taluma | LVC | 0 | 92.57 | 0.00 | |
| Colombia | Taluma | LVC | 2 | 125.21 | 3.20 | |
| Colombia | Taluma | LVC | 5 | 59.79 | 0.00 | |
| Colombia | Taluma | LVC | 7 | 1.43 | 0.50 | |
| Colombia | Taluma | AVC | -1 | 114.95 | 0.01 | |
| Colombia | Taluma | AVC | 0 | 98.12 | 0.01 | |
| Colombia | Taluma | AVC | 2 | 166.16 | 0.01 | |
| Colombia | Taluma | AVC | 5 | 57.03 | 0.35 | |
| Colombia | Taluma | AVC | 7 | 6.63 | 0.01 | |
| Brazil | Rio Grande do Sul | LVC | -1 | 34.99 | 0.00 | |
| Brazil | Rio Grande do Sul | LVC | 0 | 132.13 | 8.64 | |
| Brazil | Rio Grande do Sul | LVC | 2 | 98.38 | 4.94 | |
| Brazil | Rio Grande do Sul | LVC | 7 | 188.12 | 3.29 | |
| Brazil | Rio Grande do Sul | LVC | 14 | 100.44 | 16.47 | |
| Brazil | Rio Grande do Sul | LVC | 20 | 62.98 | 15.64 | |
| Brazil | Rio Grande do Sul | AVC | -1 | 31.70 | 7.00 | |
| Brazil | Rio Grande do Sul | AVC | 0 | 84.80 | 2.47 | |
| Brazil | Rio Grande do Sul | AVC | 2 | 91.79 | 4.94 | |
| Brazil | Rio Grande do Sul | AVC | 7 | 98.79 | 4.94 | |
| Brazil | Rio Grande do Sul | AVC | 14 | 76.15 | 6.59 | |
| Brazil | Rio Grande do Sul | AVC | 20 | 76.98 | 12.35 | |
| Argentina | Balcarce | LVC | -1 | 48.51 | 25.76 | |
| Argentina | Balcarce | LVC | 0 | 99.72 | 21.33 | |
| Argentina | Balcarce | LVC | 1 | 208.86 | 12.88 | |
| Argentina | Balcarce | LVC | 2 | 194.04 | 10.47 | |
| Argentina | Balcarce | LVC | 22 | 24.26 | 8.86 | |
| Argentina | Balcarce | AVC | -1 | 76.81 | 12.88 | |
| Argentina | Balcarce | AVC | 0 | 91.63 | 9.66 | |
| Argentina | Balcarce | AVC | 1 | 184.61 | 22.94 | |
| Argentina | Balcarce | AVC | 2 | 258.72 | 23.35 | |
| Argentina | Balcarce | AVC | 22 | 24.26 | 10.06 | |
| Argentina | Manfredi | LVC | -1 | ---- | 7.20 | |
| Argentina | Manfredi | LVC | 0 | ---- | 19.20 | |
| Argentina | Manfredi | LVC | 2 | ---- | 12.10 | |
| Argentina | Manfredi | LVC | 6 | ---- | 62.80 | |
| Argentina | Manfredi | LVC | 14 | ---- | 64.70 | |
| Argentina | Manfredi | LVC | 21 | ---- | 73.80 | |
| Argentina | Manfredi | AVC | -1 | ---- | 10.30 | |
| Argentina | Manfredi | AVC | 0 | ---- | 36.90 | |
| Argentina | Manfredi | AVC | 2 | ---- | 15.20 | |
| Argentina | Manfredi | AVC | 6 | ---- | 104.00 | |
| Argentina | Manfredi | AVC | 14 | ---- | 56.10 | |
| Argentina | Manfredi | AVC | 21 | ---- | 37.20 | |
| Trinida&Tobago | St. Augustine | LVC | -1 | 124.74 | 27.72 | |
| Trinida&Tobago | St. Augustine | LVC | 0 | 69.30 | 20.79 | |
| Trinida&Tobago | St. Augustine | LVC | 1 | 97.02 | 20.79 | |
| Trinida&Tobago | St. Augustine | LVC | 2 | 83.16 | 27.72 | |
| Trinida&Tobago | St. Augustine | LVC | 4 | 48.51 | 62.37 | |
| Trinida&Tobago | St. Augustine | LVC | 7 | 62.37 | 55.44 | |
| Trinida&Tobago | St. Augustine | LVC | 15 | 48.51 | 48.51 | |
| Trinida&Tobago | St. Augustine | AVC | -1 | 117.81 | 41.58 | |
| Trinida&Tobago | St. Augustine | AVC | 0 | 76.23 | 20.79 | |
| Trinida&Tobago | St. Augustine | AVC | 1 | 194.04 | 20.79 | |
| Trinida&Tobago | St. Augustine | AVC | 2 | 76.23 | 27.72 | |
| Trinida&Tobago | St. Augustine | AVC | 4 | 48.51 | 27.72 | |
| Trinida&Tobago | St. Augustine | AVC | 7 | 117.81 | 41.58 | |
| Trinida&Tobago | St. Augustine | AVC | 15 | 90.09 | 20.79 | |

**Supplementary Table S3. Soil mineral nitrogen dynamics at six study sites**


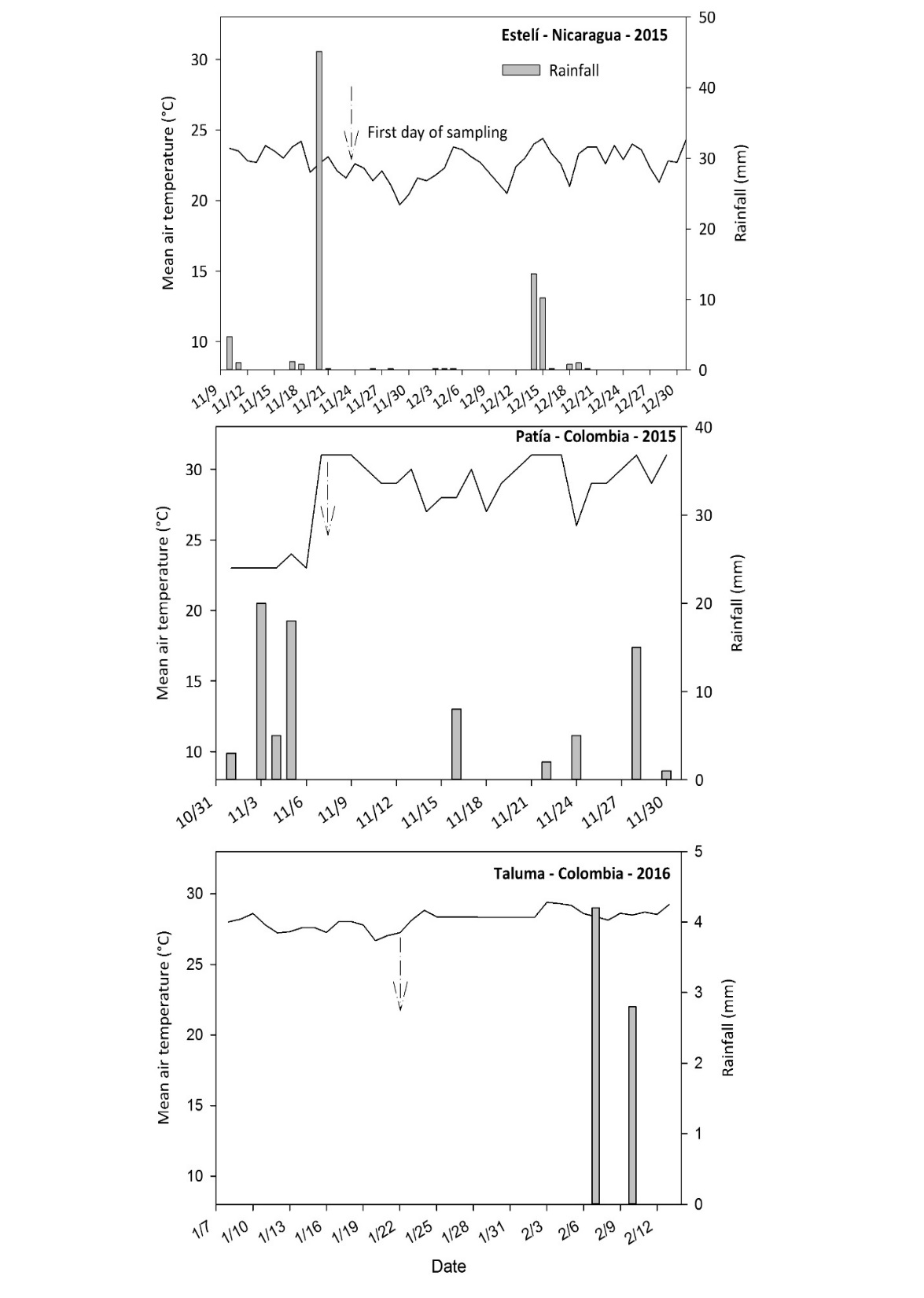

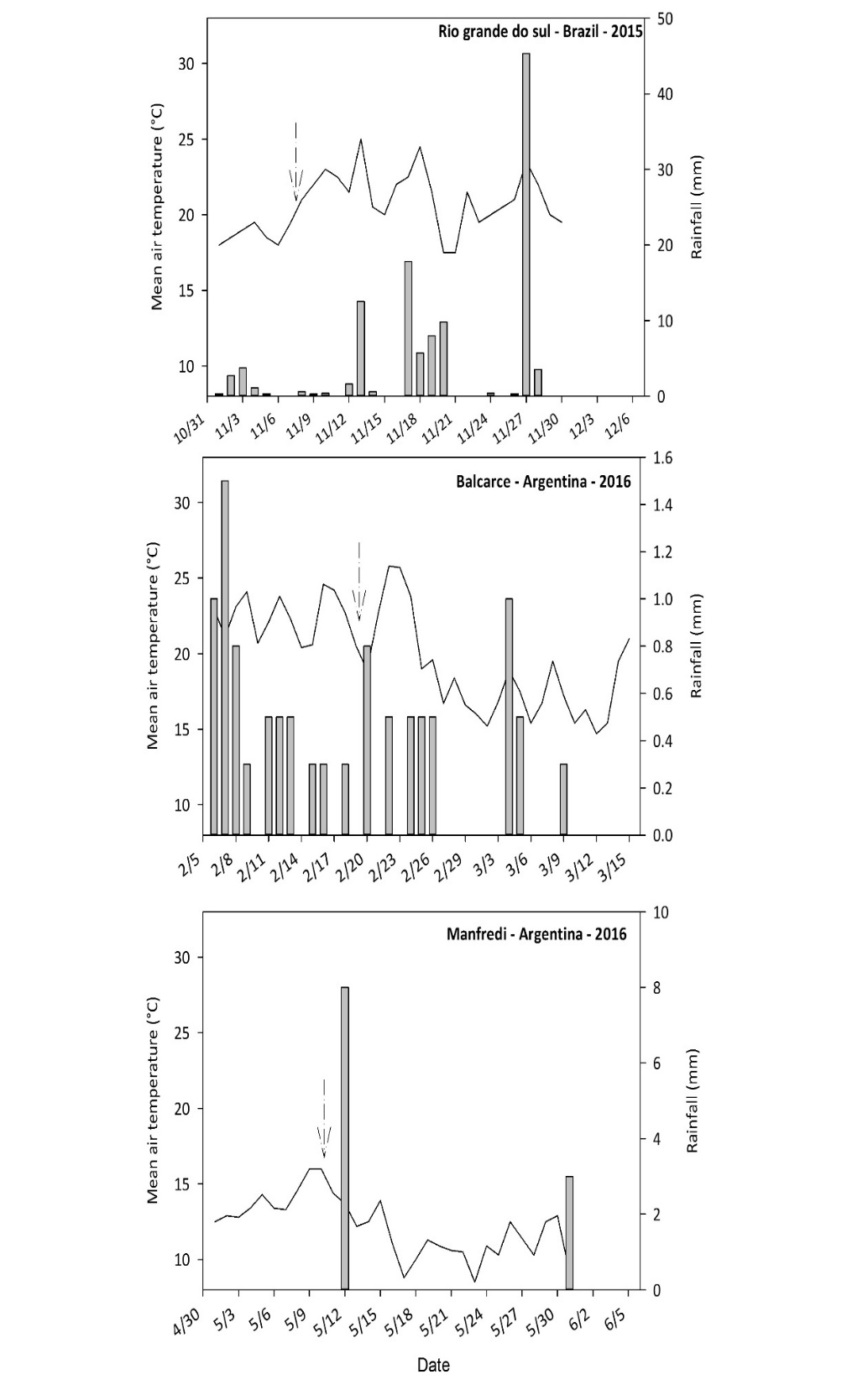

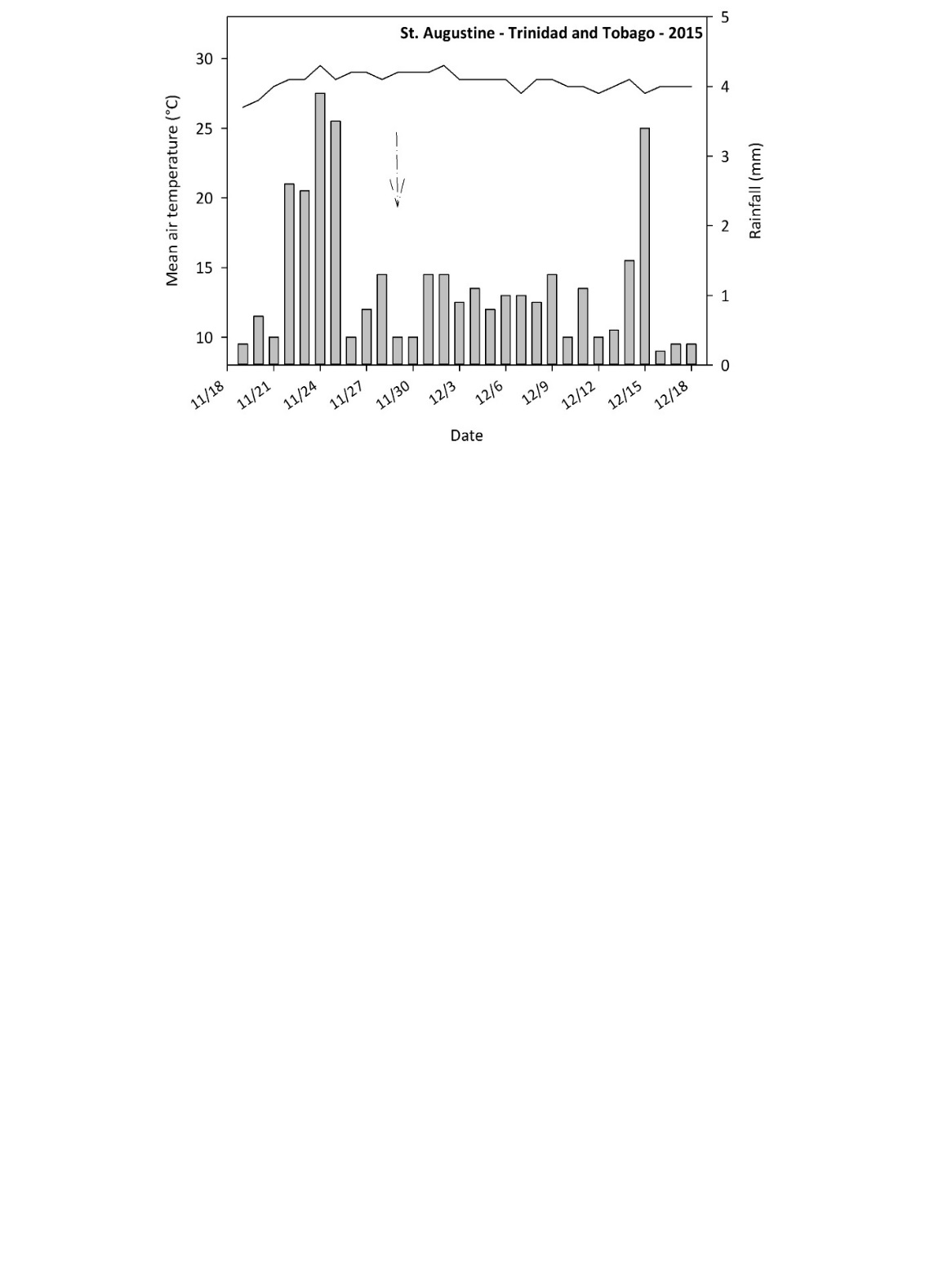


**Supplementary Figure S1**


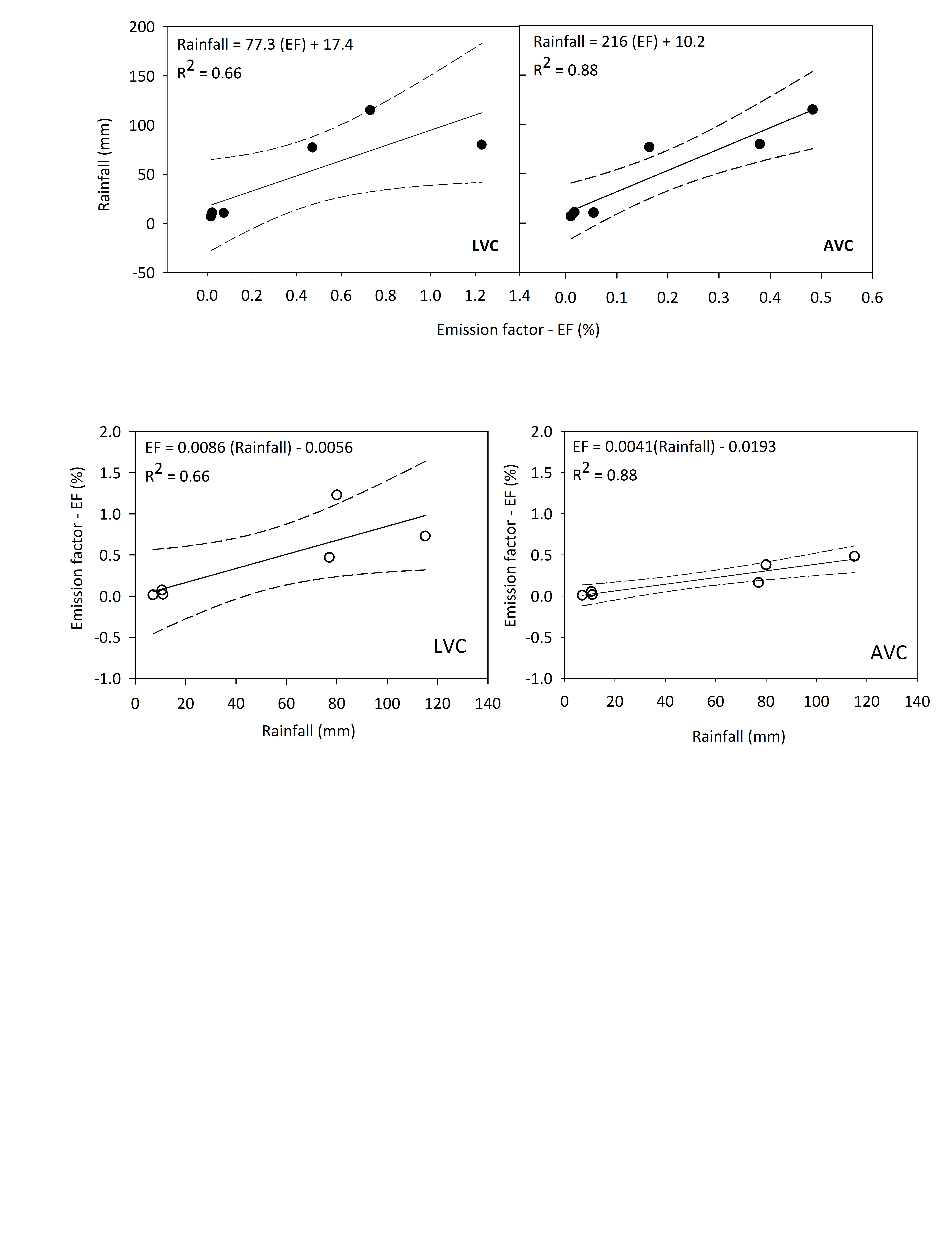


**Supplementary Figure. S2**

**Supplementary Figure. S3**


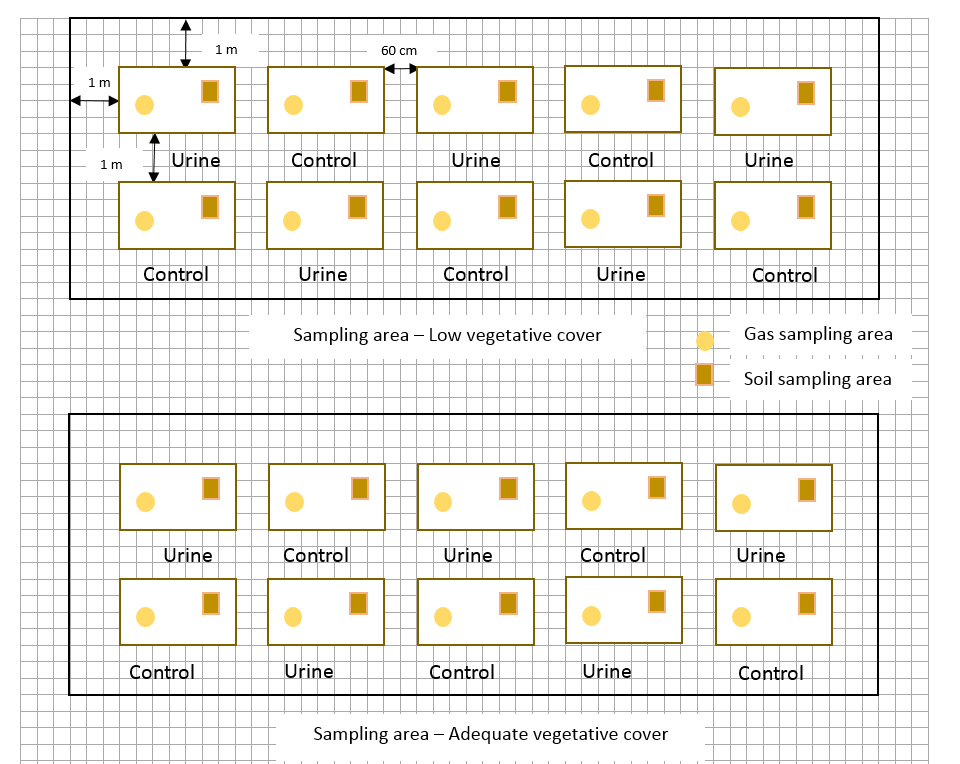


Scale 1:100 centimeters

**Supplementary Figure. S4**

**References**

1. Cruz, P. et al. Leaf traits as functional descriptors of the intensity of continuous grazing in native grasslands in the south of Brazil. Rangeland Ecology & Management **63**, 350-358 (2010).

2. da Trindade, J. K., Neves, F. P., Pinto, C. E., Bremm, C., Mezzalira, J. C., Nadin, L. B., Genro, T. C. M., Gonda, H. L., Carvalho, P. C. F., Daily forage intake by cattle on natural grassland: response to forage allowance and sward structure. Rangeland Ecology and Management **69**, 59–67 (2016).

3. ArcGIS 9.1. The mapping and analytics platform. Environmental Systems Research Institute. Redlands, California, United States URL [www.esri.com](http://www.esri.com/). (2005)
